# Animal emotions and consciousness: a preliminary assessment of researchers’ perceptions and biases and prospects for future progress

**DOI:** 10.1098/rsos.241255

**Published:** 2024-11-13

**Authors:** Matthew N. Zipple, Caleb Hazelwood, Mackenzie F. Webster, Marcela E. Benítez

**Affiliations:** ^1^ Laboratory for Animal Social Evolution and Recognition, Department of Neurobiology and Behavior, Cornell University, Ithaca, NY 14853, USA; ^2^ Department of Philosophy, Duke University, Durham, NC 27701, USA; ^3^ Department of Psychology, Emory University, Atlanta, GA 30322, USA; ^4^ Department of Anthropology, Emory University, Atlanta, GA 30322, USA

**Keywords:** animal emotion, consciousness, experimental philosophy, animal affect

## Abstract

Scientists and philosophers have long struggled with the question of whether non-human animals experience emotions or consciousness. Yet, it is unclear where the scientific consensus on these topics lies today. To address this gap, we administered a survey of professional animal behaviour researchers to assess perceptions regarding (i) the taxonomic distribution of emotions and consciousness in non-human animals, (ii) respondents’ confidence in this assessment, and (iii) attitudes towards pitfalls and potential for progress when addressing these questions. Respondents (*n* = 100) ascribe emotionality and consciousness to a broad swath of the animal taxonomy, including non-human primates, other mammals, birds and cephalopods. Respondents’ attribution of these phenomena was strongly associated with their confidence in their assessments (*R*^2^ > 0.9), with respondents assuming an absence of emotions and consciousness when they were unsure. We also identify an emergent consensus of the components involved in a functional definition of emotions. Researchers are optimistic that tools either currently exist or will exist in the future to rigorously address these questions (>85%) and that animal behaviour, as a field, should do more to encourage research works on emotions (>70%). We discuss implications for publication bias and future work in this area as well as ethical considerations regarding animal care and use.

## 1. Introduction

“I have chosen bats instead of wasps or flounders because if one travels too far down the phylogenetic tree, people gradually shed their faith that there is experience there at all.” - Thomas Nagel

Do animals experience emotions? For centuries, this seemingly simple question has been debated by philosophers and scientists, with some suggesting that emotions are uniquely human traits and others arguing that emotions play a significant role in how animals behave [1–4]. For example, Aristotle and Hume believed that animals and humans share similar desires and emotions, with Hume going so far as to extend thought and reason to non-human animals [1,3]. On the other hand, the French philosopher Descartes challenged these beliefs, arguing that animals were mere machines, lacking the capacity for emotions or consciousness [2]. The American philosopher Thomas Nagel argued that many animals clearly have their own subjective experiences, but paired this assumption with an epistemic claim that those emotions and mental states ‘may be permanently denied to us by the limits of our nature’ and therefore make poor subjects of scientific study [4].

In the nineteenth and twentieth centuries, scientific discourse on the question of animal emotions paralleled this long-running philosophical dispute, with evolutionary biologists, behaviourists and ethologists debating both the presence of emotions in animals and our ability as scientists to critically evaluate a fundamentally ‘private phenomenon’ [5]. Darwin displayed no compunction about ascribing emotions to animals, arguing in *The Expression of the Emotions in Man and Animals* that animals display and express many of the same emotions that humans do [6]. In *The Descent of Man, and Selection in Relation to Sex*, Darwin claimed that ‘the lower animals, like man, manifestly feel pleasure and pain, happiness, and misery’ [7]. But within 50 years of Darwin’s work, behaviourist theories had come to dominate animal behaviour research. John B. Watson and B.F. Skinner, among others, frowned at the notion of studying animal (and human) emotions because they considered it unscientific. For behaviourists, only behaviour—specifically behaviour as a response to external stimuli—‘counted’ as observable and quantifiable data. They argued that even if subjective experiences, like emotions, did exist in non-human animals, emotions made poor study questions because they were unobservable, unmeasurable, and unverifiable [8]. This sentiment can perhaps be best summed up by the evolutionary biologist George Williams [5] who wrote, ‘I am inclined merely to delete it [the mental realm] from biological explanation, because it is an entirely private phenomenon, and biology must deal with the publicly demonstrable’ [5].

One of the issues that plagues all researchers of emotion, and animal emotion researchers in particular, is an inconsistency in definitions across fields. Indeed, one argument for why our understanding of emotions has lagged behind other areas of cognition is that there remains fundamental disagreement on the definition of emotion [9]. This hurdle is particularly large for animal researchers because many definitions of emotions include the subjective aspect as a fundamental part of emotional experience—something that is difficult, if not impossible, to operationalize in a non-human animal. Frans de Waal neatly summarized the dilemma animal researchers face, noting that while emotions in animals are rarely denied, their importance is often questioned leaving us ‘with the curious situation that a widely recognized aspect of animal behaviour is deliberately ignored or minimized’ [10].

Despite these hurdles, research in recent decades demonstrates that, at minimum, animals experience changes in their core affective states, categorized by a spectrum of valence and arousal levels [11,12]. These conclusions come from advances in the fields of psychology, ethology, animal cognition, neuroscience and behavioural endocrinology, that have allowed researchers to monitor and examine internal states in animals. For example, comparative psychologists and neuroscientists have continued Darwin’s approach to studying animal facial expressions, now with the added scientific power of machine learning and neurobiological recordings [13,14]. Neuroscientists have focused on identifying the complex relationship between emotions and neural structures of the brain, highlighting several areas of the cerebral cortex and subcortical structures (e.g. amygdala) that can initiate and regulate emotions. These regions appear homologous in some non-human animal species, suggesting that they likely play a similar role (reviewed in [15]). These discoveries and others have opened the door for behavioural ecologists to explicitly incorporate animal emotions into theories of why and how animals do what they do. For example, Crump *et al*. argue that animal emotions and mood play a critical role in explaining winner and loser effects in animal contests [16].

The distribution of expert opinions regarding the presence and importance of animal emotions has fluctuated substantially over time, so where are we now? In this paper, we set out to quantify the current distribution of expert thought on these topics by surveying animal behaviour researchers across different fields and academic stages. The main goals of this study are to quantify researchers’ current positions on animal emotions in general (‘do animals have emotions? how confident are you in that assessment?’), the taxonomic distribution of animal emotions (‘which animals have emotions?’), and whether these questions are perceived as answerable (‘do we have the tools to study animal emotions?’). By making clear the current distribution of thought, we hope to instigate interdisciplinary conversation and help guide future research initiatives in this growing field.

When claiming that animals do or do not have emotions, we are making a philosophical claim that depends on the operative concept of ‘emotion’ we have in mind. Different researchers have different criteria for what emotions are in the first place. In such circumstances, researchers studying the exact same model system and working with the exact same empirical data may produce opposite verdicts about the presence and importance of animal emotions. To investigate this philosophical question, we rely on a methodology referred to as ‘experimental philosophy’. Experimental philosophy combines the conceptual frameworks traditionally associated with philosophy and the experimental rigour of cognitive science [17]. It uses survey data to generate understanding about the concepts that scientists use—concepts that are instrumental for their research—instead of legislating those concepts from the armchair (see similar approaches in psychology; e.g. [18]). In biology, these methods have recently been used to examine differences in how geneticists define genes [19–21], as well as biologists’ positions on ‘innateness’ [22,23] and evolutionary and ecological models [24].

To our knowledge, there have been no previous attempts to quantify the distribution of thought regarding animal behaviour researchers’ perceptions of the taxonomic distribution of animal emotionality. Previous studies have focused on public opinions of animal emotions revealing that people assign declining empathetic capacity to animals with increased phylogenetic distance to humans [25]. People are also more likely to empathize with and show compassion towards species that are more closely related to humans [26–29], with implications for conservation [30,31]. Whether these same assessments are shared by scientific experts remains unknown. Understanding experts’ opinions is important, both because such opinions are most informed by empirical insights and because experts’ opinions, biases, and research interests provide insight into the direction that a field of study will go in the future. Here, we take the first step towards filling this gap by conducting a survey of professional animal behaviour researchers in which we ask them to describe their beliefs about the taxonomic distribution of animal emotions and consciousness and their confidence in those assessments. The result is the first assessment of how animal behaviour researchers, as a group, think about the importance of emotions and consciousness in non-human animals.

## 2. Methods

In this study, we collected survey data from professional animal behaviour researchers who provided responses to multiple-choice questions, free-form text fields and Likert scales [32]. Responses were solicited through two primary means. First, we emailed administrators in all departments on US News and World Reports’ list of best graduate school programmes in behavioural neuroscience, cognitive psychology, ecology/evolutionary biology and neuroscience/neurobiology, requesting that our survey be distributed to relevant members of their department. Second, we posted solicitations of our survey on Twitter (now X), explaining the type of respondents that we were seeking and providing a basic overview of the types of questions respondents would be asked (see electronic supplementary material for the full text of solicitations).

With this sampling approach, we cannot rule out the possibility of response bias owing to individuals self-selecting into participation in the survey. As such, we assume that our respondents are representative of animal behaviour researchers who have relatively strong interest in or experience with the questions in our survey, though perhaps not representative of all animal behaviour researchers as a group. When completing our survey, respondents first provided individual data about their career stages, research disciplines, and study system. With these data, we further examined (and ruled out, see §3) potential biases due to career stage or field.

Respondents answered a series of questions about whether they ascribe emotional responses and consciousness to various taxa, the relevance of various factors (such as phylogenetic proximity to humans, social behaviour, group living, facial expressions, etc.) in shaping their answers, and their confidence in their assessments. One of our goals was to elicit definitions of emotions from researchers, and we thus did not include a definition ourselves. We did not have this same goal regarding consciousness, so we defined consciousness for respondents, using a ‘self-consciousness’ or ‘self-awareness’ definition (an animal being aware of its own existence [33–35]).

Finally, we surveyed the participants’ meta-level beliefs about the role of emotions in their discipline: Does talk of animal emotions risk anthropic fallacies? Should research into non-human animal emotions be encouraged? Does the participant feel comfortable voicing their opinions about non-human animal emotions in professional settings? We have included the full survey in the electronic supplementary materials.

### 2.1. Inclusion criteria

We included in our analyses only respondents who (i) identified as professional scientists or current academic students and (ii) navigated all the way to the end of the survey. As a basic test of an individual carefully considering the questions and giving good-faith responses, we included only respondents who (iii) identified either ‘all or nearly all’ or ‘most’ humans as displaying emotions.

### 2.2. Statistical analyses

We performed all statistical analyses in R (version 4.2.2) [36]. For mixed effects models, we used the package glmmTMB (version 1.1.5) [37]. We created Likert scale visualizations using the ‘likert’ package (version 1.3.5) [38]. Details on model structure, including random and fixed effects are provided in §3.

### 2.3. Ethical approval

Because we did not store sensitive personal information and our survey did not pose a risk of adverse outcomes for participants, we obtained institutional review board exemptions from each of our institutions (Cornell University, Duke University and Emory University).

## 3. Results

### 3.1. Respondent characteristics

In total, we received 100 complete responses to our survey that met the inclusion criteria. Forty-five per cent of respondents were graduate students, 20% were postdocs and 31% were faculty (the remainder fell into other professional categories; see table 1). Most respondents identified as belonging to multiple fields and most studied multiple taxa. Our sample included a large number of behavioural ecologists (63%), evolutionary biologists (38%) and neuroscientists (26%), as well as a smaller number of biological anthropologists (13%), cognitive psychologists (11%) and biological psychologists (12%). The most common taxa of study among respondents were birds (43%), non-human primates (32%), and other mammals (48%), though each of the taxa that we asked respondents to assess were studied by at least some members of our sample (table 1). We were able to assess respondents’ gross geographic distribution by looking at the country domain from their email addresses (though we did not link email addresses to other responses). Of our 100 respondents, 69 originated from North or Central America, 21 were European in origin and the remaining 10 originated from Asia, South America and Africa.

**Table 1.** Academic traits of included respondents to our survey.

		percentage of respondents (*n* = 100)
***career stage***		
	graduate student	45
	postdoctoral researcher	20
	assistant professor or equivalent	13
	associate professor or equivalent	10
	full professor or equivalent	5
	emerita	2
	undergraduate student	2
	other PhD researcher or adjunct faculty	3
***self-identified fields of study***		
	behavioural ecologist	63
	evolutionary biologist	38
	biological anthropologist	13
	neuroscience	26
	cognitive psychology	11
	biological psychology	12
	other	26
***self-identified taxa of study***		
	non-human primates	32
	other mammals	48
	birds	43
	amphibians or reptiles	11
	fish	15
	squids, octopus or cuttlefish	2
	insects	16
	other invertebrates	8

### 3.2. Assessing potential response bias introduced by career stage or field of study

We did not weight our survey responses to try to match characteristics of the population of animal behaviour researchers. To assess the potential for an over- or under-represented subgroup of respondents to skew our results, we performed two simple analyses. We tested for differences in respondents’ assessments of the presence of emotions and consciousness in various taxa depending on two salient characteristics—their career stage and field of study. We found that neither career stage (‘student or postdoc’ versus ‘faculty’) nor field of study (‘neuroscience’ versus ‘all others’) predicted respondents’ assessments of the taxonomic breadth of emotions or consciousness, or their confidence in these assessments (*p* > 0.1 in all cases; see electronic supplementary material, figure S1). These results increase our confidence that any potential response biases in our sample do not appear to be primarily driven by career stage or field of study.

### 3.3. Perceived taxonomic distribution of emotions in non-human animals

**Figure 1.**
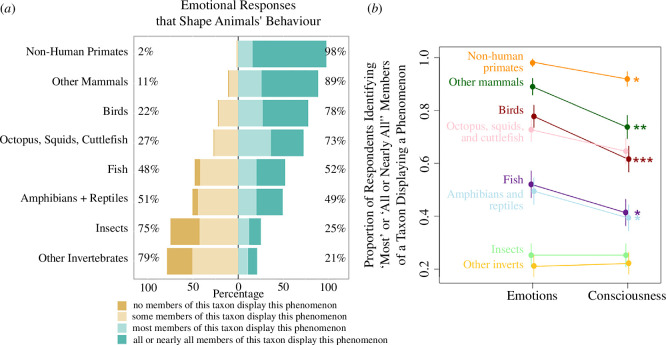
(*a*) Respondents’ assessment of the distribution of emotional responses that shape animals’ behaviour across a range of non-human taxa. (*b*) For most taxa assessed, respondents were more likely to ascribe emotional responses to animals than they were consciousness. Asterisks represent significance: *p < 0.05; **p < 0.01; ***p < 0.001.

Overall, animal behaviour researchers identify a wide taxonomic breadth of animals as experiencing emotions that shape their behaviour. Majorities of those surveyed ascribed emotions to ‘most’ or ‘all or nearly all’ non-human primates (98%), other mammals (89%), birds (78%), octopus, squids and cuttlefish (72%) and fish (53%). And for all taxa considered, a majority of researchers ascribe emotions to at least some members of each taxon, including insects (67%) and other invertebrates (71%). We present our full results in figure 1.

### 3.4. Perceived taxonomic distribution of consciousness in non-human animals

Animal behaviour researchers also ascribed consciousness to a broad taxonomic breadth of animals, although a more restricted range as compared with emotions. As with emotions, majorities ascribed consciousness to ‘most’ or ‘all or nearly all’ non-human primates (92%), other mammals (73%), octopuses, squids and cuttlefish (64%) and birds (61%). As with emotions, most researchers ascribe consciousness (electronic supplementary material, figure S2) to at least some members of each taxon, including insects (51%) and other invertebrates (52%).

Each respondent assessed the frequency of emotions and consciousness across multiple taxa. Combining each of these responses into a mixed effects model with random effects of respondent ID and taxon ID revealed that respondents were substantially more likely to ascribe emotions to most or all members of a taxa than they were to ascribe consciousness to the same (*p* < 0.0001). When breaking these differences down within different taxa (figure 1), respondents were more likely to ascribe emotions than consciousness to non-human primates (*p* = 0.047), other mammals (*p* = 0.002), birds (*p* = 0.0009), fish (*p* = 0.02) and amphibians and reptiles (*p* = 0.04).

### 3.5. Respondents’ confidence in their assessments of animals’ emotions and consciousness

**Figure 2.**
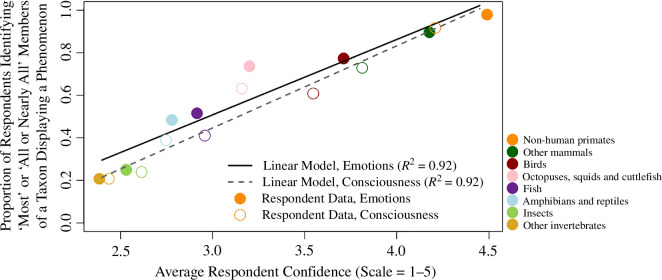
Respondents’ assessment of the distribution of emotions or consciousness in a given taxon was almost perfectly predicted by how confident they were in that assessment. For each taxon, respondents provided their confidence in their assessment 1 (‘not at all confident’) to 5 (‘certain’). For both assessments of emotions (*R*^2^ = 0.92) and consciousness (*R*^2^ = 0.92), respondents ascribed phenomena more broadly within a taxon when they were more confident in their assessment.

Respondents displayed a wide range of confidence in their assessment of the distribution of emotions and consciousness, depending on the taxon in question (figure 2). Respondents were most confident in their assessments of non-human primates (mean = 4.5 on a 1–5 scale) and other mammals (mean = 4.2), followed by birds (mean = 3.7). Respondents’ confidence declined when considering ectotherms, with respondents less confident about their assessment of cephalopods (mean = 3.2), followed by fish (mean = 2.9), amphibians and reptiles (2.8), insects (2.5) and finally other invertebrates (mean = 2.4). Confidence regarding assessments of consciousness followed an identical taxonomic pattern as did confidence in assessment of emotions. Generally, confidence in the assessment of the two phenomena was quite similar (note mostly overlapping 95% confidence intervals in electronic supplementary material, figure S3).

The concordance between respondents’ assessment of the distribution of emotions and consciousness in a given taxon and their confidence in that same assessment is striking—as a group, respondents’ average confidence in their assessment of emotionality for a given taxon almost perfectly predicted the distribution of emotions of that they ascribed to that taxon (*R*^2^ = 0.92, *p* = 0.001, figure 2).

### 3.6. Biases shaping researchers’ assessments of animal emotions

To examine potential biases shaping responses, we asked respondents to think about their responses and assess how important various animal characteristics were in shaping those assessments (‘not at all important’, ‘somewhat important’, ‘very important’). We then asked respondents to assess the extent to which those same characteristics were necessary for an animal to experience emotions (‘not at all necessary’, ‘somewhat necessary’, ‘essential’). Thus, respondents were given the opportunity to provide answers that were logically at odds with each other, indicating a bias in scientists’ thinking on these topics. Specifically, respondents could identify a characteristic as being ‘not at all necessary’ for an animal to experience emotions, while still identifying these same characteristics as ‘somewhat’ or ‘very’ important to their answers.

**Figure 3.**
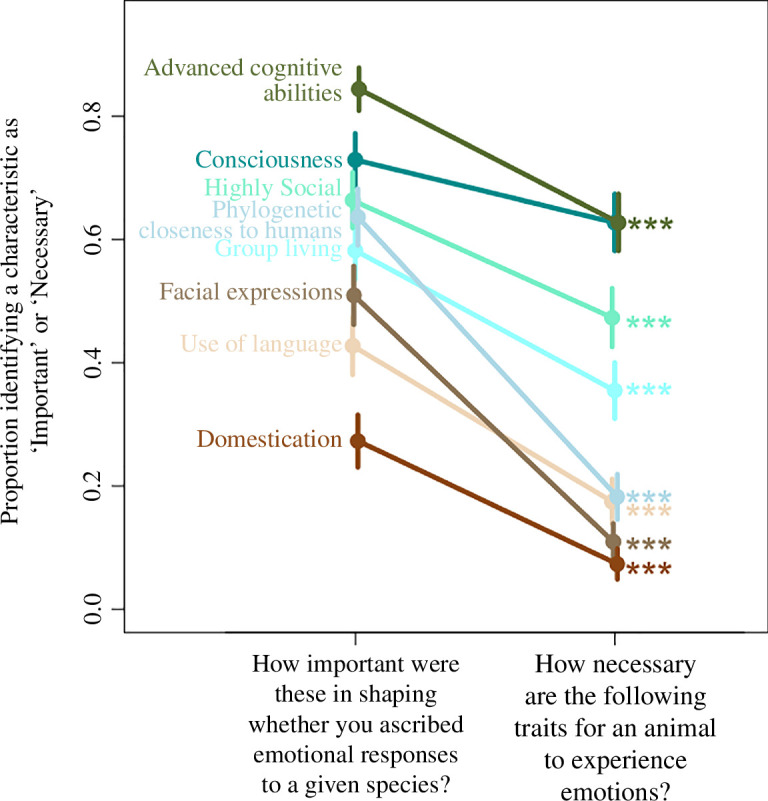
A mismatch exists in respondents’ sense of whether a given trait is necessary for animals to experience emotion (right) and their own assessment of how important that same characteristic was in shaping whether they ascribed emotional responses to a given species (left). Respondents’ assessments were shaped by many traits that they admitted were not necessary for an animal to experience emotions. Asterisks represent significance: ***p < 0.001.

For nearly every characteristic that we asked about, we detected a discrepancy between what respondents identified as important (‘somewhat’ or ‘very’) in shaping their assessments of whether animals experience emotions and what they identified as necessary (‘somewhat necessary’ or ‘essential’) for animals to experience emotions (figure 3). Remarkably, two characteristics (facial expressions and phylogenetic closeness to humans) were identified by large majorities of respondents as not at all necessary (91% and 81%, respectively) but were also identified by majorities (51% and 68%) as at least somewhat important in shaping their assessments. Sizable minorities also identified domestication (25%) and use of language (44%) as important in shaping their assessments even though many fewer identified these same characteristics as at all necessary for animals to experience emotions (6% and 15%, respectively). Even when large portions or majorities of respondents identified a given characteristic as necessary for animals to experience emotions, even more respondents identified these characteristics as important in shaping their thinking, including group living (34% somewhat necessary or essential versus 60% somewhat or very important), being highly social (46% versus 67%), and advanced cognitive abilities (64% versus 87%).

The one characteristic that respondents identified as both important and necessary without a significant difference (*p* > 0.05) in their assessment was consciousness. Seventy-five per cent of respondents identified animals’ consciousness as important in shaping their view of whether those animals had emotions, and 65% said that consciousness was at least somewhat necessary for animals to experience emotion. This latter pattern mirrors the tight perceived taxonomic linkages between emotions and consciousness that respondents had previously expressed (figure 1).

### 3.7. Perceptions of the risk of anthropic fallacies

**Figure 4.**
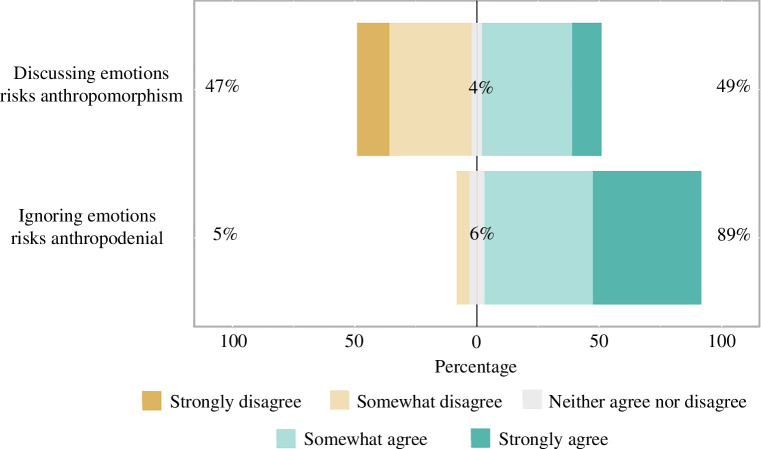
Respondents are divided on whether discussing animal emotions risks inappropriately anthropomorphizing animals’ experiences. Respondents generally agree, however, that ignoring the possible existence of animal emotions risks anthropodenial.

Respondents were divided on the topic of the risks posed by the twin anthropic fallacies of anthropomorphism and anthropodenial [39,40]. Overall, 49% agreed that ‘Discussing the role of emotions in non-human animals’ behaviour risks inaccurately projecting human experience onto study subjects (i.e. anthropomorphizing)’, while 46% disagreed (5% neither agreed nor disagreed). Respondents had a more universal view regarding anthropodenial, with 89% of respondents agreeing that ‘Failing to consider the role of emotions in non-human animals’ behavior risks ignoring homologous mechanisms that are involved in both human and non-human behavior (i.e. anthropodenial)’ and only 6% disagreeing (5% neither agreed nor disagreed). Full results are displayed in figure 4.

Among respondents who addressed both potential anthropic fallacies (*n* = 98), 39% agreed that both anthropomorphism and anthropodenial pose a risk. Another 46% agreed that anthropodenial is a risk, but disagreed that anthropomorphism is. Only 5% felt the opposite, that anthropomorphism is a risk, but not anthropodenial (the remaining 10% selected ‘neither agree nor disagree’ for at least one statement). Thus, nearly all researchers identify animal emotions as an area where one or more anthropic fallacies pose a risk to scientific progress, with a substantial minority (39%) identifying both issues as conflicting concerns.

### 3.8. Perceptions of scientists’ abilities to measure emotions and consciousness in non-human animals

Based on the philosophical debate that we outline in §1, it seemed possible that some respondents might argue that the question of whether a given animal has an emotional or conscious life might be untestable. To assess this possibility, we asked respondents to state whether they believed that animals’ emotions and consciousness, separately, (i) could be measured using existing techniques, (ii) could not be measured using existing techniques, but given reasonable advances in techniques would be measurable in the future, or (iii) could not be measured using existing techniques, nor would it be possible to do so given reasonable advances in the future (see note in electronic supplementary material about selecting multiple options).

Only 11% of respondents said that animal emotions could not be measured now nor could they be reasonably expected to be measurable in the future. In contrast, 38% of respondents said that animal emotions can currently be measured with existing techniques, and another 52% said that although not currently possible, measuring animal emotions would become possible in the future given reasonable advances in techniques. Responses were essentially identical with regards to measuring consciousness (15% not possible now or in the future, 36% possible now, 49% not possible now but will be in the future).

### 3.9. Perceptions of the field of animal behaviour’s treatment of animal emotions

A large majority of respondents agreed that they would feel comfortable describing their beliefs regarding animal emotions and consciousness at a professional meeting (74%), while only 12% disagreed (the remainder neither agreed nor disagreed). Those who strongly agreed with the statement that they would be comfortable doing so were more confident in their average assessment of the presence or absence of emotions and consciousness in a given taxon (mean confidence rating = 3.6 for ‘strongly agree’, 3.0 for ‘somewhat agree’, 2.9 for other responses; ‘strongly agree’ versus other categories *p* < 0.0002).

A large majority (71%) of respondents also agreed that the field of animal behaviour ‘should do more to encourage research into non-human animal emotions, feelings, and consciousness’ while only 12% disagreed. At the same time, only a slight majority (51%) of respondents agreed that the field of animal behaviour already encourages researchers to consider animal emotions, while 35% disagreed.

### 3.10. Animal researchers’ definitions of emotion

As the final question of our survey, we asked respondents to define emotion. After reading respondents’ answers, two of us (M.N.Z. and M.F.W.) independently classified respondents’ answers as containing each of four components that many answers had in common: (i) reference to emotion resulting from internal and/or external stimuli, (ii) reference to the function of emotion as motivating behaviours, (iii) reference to subjective experience of an emotion/affective state or references to ‘mentality’, ‘consciousness’, ‘state(s) of mind’, or ‘feeling(s)’, and (iv) reference an explicitly social, communicative function of expressing internal state to others. These two independent assessments closely matched each other (>84% agreement for an exact match of all components; for each of the four components Cohen’s *K* = {0.85, 0.92, 0.70, 0.88}). M.N.Z. and M.F.W. then collaborated to come to an agreement on responses that they had characterized differently.

Eighty-one respondents out of the 100 included in the broader survey provided a definition of emotions, perhaps indicating that for a substantial subset of respondents verbalizing their working definition was a challenge. Of the 81 definitions of emotion that respondents provided, 80 contained at least one of the components described above, with a slight majority (41/80) containing multiple components.

**Figure 5.**
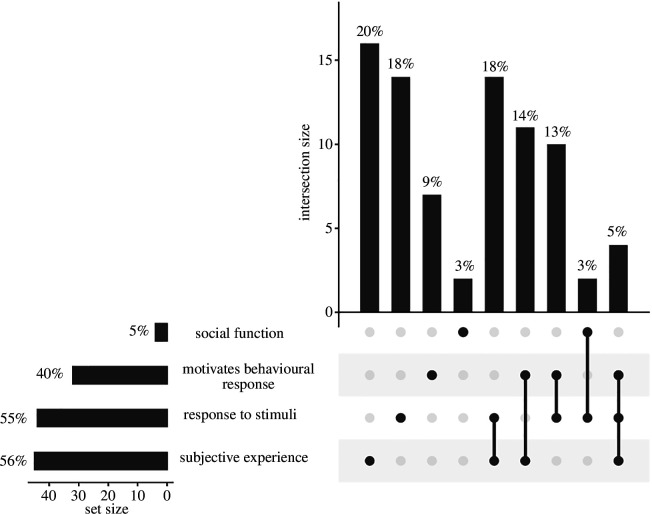
The distribution of components that respondents included in an open-ended request for their definition of emotions. Each vertical bar represents the percentage of respondents that included a given combination of potential elements that we scored in their definition. These components emerged organically after reading respondents’ definitions, rather than being pre-ordained. Horizontal bars represent the total percentage of respondents that included each individual element in their definition (rounding may result in apparent differences between horizontal bars and sums of vertical bars).

A majority of definitions contained a reference to emotions being a response to either internal or external stimuli (55%). A majority contained a reference to emotions being subjective experiences or otherwise containing words related to consciousness or mindedness (56%). A substantial minority identified emotions as functioning to motivate behaviours (40%). We note that earlier in our survey when we had asked respondents about their perceptions of the distribution of emotions, we reference ‘emotion that shape animals’ behavior’. It is possible that this framing shaped individuals’ likelihood of including this motivational aspect of emotion in their definitions, but given that this dimension of emotion was not the most common aspect of respondents’ definitions, any such unintended influence appears to have been limited in scope. A full breakdown of the distribution of these components and their overlap can be seen in figure 5.

Despite substantial variation in the working definitions of emotions that respondents provided, individuals were no more or less likely to ascribe emotions to animals based on whether they included or excluded a given component. To assess this relationship while limiting the number of statistical tests performed, we first summed individuals’ Likert scale responses regarding their perceptions of the presence of animal emotions in each of the eight non-human taxa that we asked about (total possible range = 8–32). We then asked whether this total index value was predicted by whether an individual included each of the four components in their working definition of emotion. We found there to be no relationship between definitional components and overall assessment of the presence of emotions in non-human animals (*t*_73_; *p* > 0.3 in all cases; electronic supplementary material, figure S4). Within each of the eight individual taxa, there was also no relationship between assessment of the distribution of emotion and individual definition components, after correcting for multiple hypothesis testing. In other words, although respondents may have had different models in their minds for what the word ‘emotion’ conveys, this did not affect their assessment of the taxonomic distribution of emotions in animals.

## 4. Discussion

In our survey, professional animal behaviour researchers described a wide range of opinions regarding animal emotions. Animal behaviour researchers ascribed both emotionality and consciousness to a broad swath of animal taxonomy and majorities identify questions regarding animal emotions as important and answerable. There were no differences in respondents’ gross-level assessment of the taxonomic distribution of emotions and consciousness depending on career stage or field of study. Several additional trends in researchers’ opinions emerge across a range of specific topics, including biases in researchers’ assessments, the relative risks of anthropomorphism and anthropodenial and prospects for future progress.

### 4.1. Taxonomic distribution of emotions and consciousness

Researchers were more likely to attribute both emotional experiences and consciousness to animals that fell phylogenetically closer to humans (figures 1 and 2). Large majorities ascribe emotions to most non-human primates, other mammals, birds and cephalopods (in that descending order). Indeed, this consistent phylogenetic trend is particularly striking given that a large majority (81%) of researchers stated that being phylogenetically close to humans was ‘not at all necessary’ for animals to experience emotions. This result closely mirrors studies of attitudes present in the general public [25,26]. Our results may also shed light on the likely cause of this phylogenetic relationship, at least in expert opinion. Respondents were dramatically less confident in their assessments of the presence of emotions and consciousness in taxa that were more phylogenetically distant from humans, and in the absence of confidence, scientists tend to assume the that these phenomena are less likely to occur (i.e. they show a conservative bias).

Respondents’ assessment of cephalopod emotionality and consciousness provided the single exception to this phylogenetic pattern. Substantial majorities of respondents identified most ‘octopus, squids, and cuttlefish’ as showing both emotions (73%) and consciousness (65%), in contrast to insects and other invertebrates. Only 20–25% of researchers identify most other invertebrates as displaying emotions or consciousness. What is the source of this difference in researchers’ perceptions regarding cephalopods? Since almost none of our respondents studied these species directly (2%), it seems unlikely that this striking deviation in assessments is primarily the result of personal experience. One possibility is that this difference stems from the institutional level, as cephalopods increasingly receive greater oversight and protection in animal research (especially in Europe) relative to other invertebrates. An alternative possibility is that researchers have been exposed to the cognitive abilities of cephalopods through both academic and popular literature and films on the topic (e.g. *Other Minds* [41] and *My Octopus Teacher* [42]). The descriptions of individual behaviour in these works may be so striking as to make a lasting impact on researchers’ assessment of the emotionality and consciousness of these animals. This possibility highlights both the powerful opportunity of charismatic portrayals of behaviour to inform scientists’ understanding of systems with which they lack direct experience, but also the potential danger of such charismatic portrayals, should they lack scientific rigour (we make no such assumption here).

Respondents were less likely to ascribe consciousness than emotionality to ‘most’ or ‘all or nearly all’ members of all vertebrate taxa that respondents considered (non-human primates, other mammals, birds, fish and reptiles and amphibians; figure 1). This consistent pattern is a bit difficult to explain, especially because 65% of respondents identified consciousness as being ‘somewhat necessary’ or ‘essential’ for animals to experience emotions (suggesting that consciousness should evolve prior to emotions).

One possible explanation for this difference is methodological—we defined consciousness as self-awareness, which may be a more restrictive definition than respondents’ working definition of emotions. We purposefully did not define emotions for respondents, but instead sought to quantify their unspoken sense of how emotions appear in non-human animals. Because of this methodological difference in assessing the two phenomena, we do not draw strong conclusions from respondents’ differential attribution of emotions and consciousness to a given taxon. We are nonetheless confident in concluding that respondents generally ascribe consciousness and emotions to the same taxa and that attribution of both phenomena declines as the phylogenetic distance from humans increases (figures 1 and 2; electronic supplementary material, S2).

### 4.2. Biases in respondents’ assessments

Respondents described many factors that had influenced their assessment of the presence of emotion in a given taxon, while also stating that those same factors were not at all necessary for an animal to experience emotions. Many of these biases appear to result from the same subconscious heuristic: researchers are most likely to identify animals as showing emotions and consciousness if they share characteristics with humans. These characteristics included facial expressions, language, advanced cognitive abilities and group living. But they also extended to characteristics not obviously associated shared by humans, including domestication (but see [43] for discussion of human self-domestication).

One of the biases that may have the greatest impact on the overall discourse regarding animal emotions and consciousness is a conservative bias in the absence of evidence. When researchers felt less certain regarding the distribution of emotions and consciousness in a given taxon, they were less likely to ascribe these phenomena to that taxon. Indeed, very few respondents expressed a high level of confidence that a taxon *lacked* emotions or consciousness. In the absence of confidence that such phenomena exist, scientists appear to assume that they do not. This attitude was summed up succinctly by one respondent who had stated that no members of any non-human taxa displayed consciousness. To explain this choice in a comment the respondent wrote, ‘As far as we know. I’m a scientist, I don’t guess’.

### 4.3. Implications of conservative bias for publication and discourse

How might the overall conservative bias that we observe shape the landscape of publications on the topic of animal emotions? Because we asked our respondents about their confidence levels, we can play out a counterfactual in which we had screened out any participants who were not confident in their assessment—responding with a confidence of 3 or lower to a given question. Such a scenario would seem like a reasonable proxy for the types of respondents that might broadcast their arguments or beliefs regarding animal emotions and consciousness in a more rigorous, non-anonymous medium, such as a scientific publication.

If we had considered only the perspectives of confident scientists, we would have reached more extreme conclusions than we did above regarding the distribution of emotions and consciousness. Such respondents ascribed emotions to ‘most’ or ‘all or nearly all’ taxa at substantially higher rates for nearly all taxa considered, including other mammals (96% for high confidence respondents versus 89% overall), birds (95% versus 78%), cephalopods (95% versus 73%), fish (81% versus 52%), amphibians and reptiles (85% versus 49%), insects (45% versus 25%), and other invertebrates (38% versus 21%).

For the questions that we asked here, our overall conclusions did not hinge on respondents’ confidence. Yet, it reveals what might otherwise be a hidden bias in the ‘consensus’ scientific view that develops on any given topic—if those that are most confident differ strongly from the median member of a field, those confident (and presumably louder) voices will be very likely to have an overweight influence on discussion both within the scientific community and in more public spheres. Indeed, respondents that were more confident in their answers said that they would feel more comfortable describing their beliefs at a professional conference.

The nature of these stronger voices has the potential to change over time. For example, among the respondents to our survey, substantially more respondents identified anthropodenial as a risk (89%) than said the same of anthropomorphism (49%). While we lack a direct survey comparison on the topic, this distribution of thought likely represents a dramatic shift from the twentieth century, when an aversion to anthropomorphism was dominant among animal behaviour researchers [5,40]. Still, nearly half of researchers stated that anthropomorphism was a concern in animal emotion research. How do we reconcile these two concerns? Future research should strive to bridge this gap to both encourage the lowering of previous barriers to research in areas related to phenomena previously assumed to be ‘private’, including mindedness, emotionality, and subjective experience, while being cautious of the very real perils of anthropomorphism.

### 4.4. Ethical implications for animal care and use

Animal scientists face both a legal and ethical imperative to consider the magnitude of animal suffering that a given scientific study will cause. Yet, reasonable people can and do disagree strongly about how much weight to give animal suffering, both physical and psychological, in this calculation. Below we explore some of the potential implications of our respondents’ perceptions of animal emotions for animal research ethics, broadly. We take no personal positions on these topics here, and may or may not agree with the consensus views that our respondents describe.

A recent high-profile study that generated substantial ethical disagreement in the animal behaviour community highlights the importance of researchers’ perceptions of animal emotions for deciding whether an experiment should be performed. A study was published in 2022 in the *Proceedings of the National Academy of Sciences* that made use of monkeys who had been the subjects of a mother–offspring separation protocol (for a different experiment) to ask whether mothers whose infants had been removed would interact with a soft monkey-like toy in the absence of their infant [44]. The separation was not conducted by the author of the paper and would have occurred in the absence of her involvement—she was simply asking whether mothers in such a circumstance would form attachments to soft, inanimate toys. Since the article’s publication, more than 260 researchers have co-authored a letter [45] in which they argued that the original experiment created so much animal suffering that any publication that made use of its existence was ‘unethical’ and ‘outdated’. The letter’s arguments centre on the psychological distress that they claim accompanies a disruption of the mother–offspring bond in primates. The authors state, for example, that primates ‘mourn’ the deaths of offspring—a word that inherently invokes a complex set of emotional and physical reactions to the death of a close social partner.

The results of our survey cannot lead to a resolution of this debate. However, they do indicate that very large majorities of animal behaviour researchers identify ‘most’ or ‘all or nearly all’ non-human primates as experiencing both emotions (98%) and consciousness (92%). It may therefore be appropriate that Institutional Animal Care and Use Committee (IACUC) includes considerations of psychological suffering in their determination of whether an experiment may be performed. Such considerations are already mandated in non-human primates via a 1985 amendment to the US Animal Welfare Act [46], but given the widespread attribution of emotions by researchers in our survey, it may be appropriate to expand the taxonomic breadth of this requirement.

The value of an institutional process that considers extreme arguments from all sides without being dictated by them is especially great in ethical areas where individual scientists disagree strongly. How then do respondents’ assessments of animal emotions and consciousness align with IACUC guidelines in the United States? Overall, consistent with the scope of IACUC oversight, respondents made substantial distinctions between the distribution of emotions and consciousness in vertebrate and invertebrate animals. For each vertebrate taxa, at least 49% of respondents identified ‘most’ or ‘all or nearly all’ members of a taxon as experiencing emotions (at least 39% said the same of consciousness). In contrast, only 21–25% of respondents said the same of insects and ‘other invertebrates’

### 4.5. Animal behaviour researchers’ definitions of ‘emotion’

Researchers provided diverse definitions of emotions that frequently included three shared elements– emotions involve an animal’s (i) response to a stimulus as a result of their (ii) subjective experience of that stimulus and (iii) that this response motivates a change in an animal’s behaviour. Although only 5% of respondents included all three of these common elements in their free-response definitions of emotions, the inclusion of these different components did not predict respondents’ assessment of the distribution of emotions across taxa.

There has been much debate in both the human and animal emotion literature regarding the proper definition of emotion [9,47–50]. The definitional elements put forth by respondents to our survey are similar to components identified by a previous survey of distinguished emotion scientists, who included both structural and functional components in their definitions [48]. For example, many of our respondents include a ‘response to a stimulus’ component in their definition of emotions, which is closely aligned with emotion researchers’ inclusion of ‘neural systems’ and ‘response systems’ as structural components of emotions. Similarly, respondents in [48] identified ‘feelings or feeling state’ as another structural component of emotions, closely matching our respondents’ attribution of a subjective experience to emotion. And, in terms of function, our respondents’ assessment that emotions motivate a change in animals’ behaviour closely matches emotion researchers’ assessment that emotion ‘recruits response systems’, ‘motivates cognition and action’, and ‘orders, organizes, and coordinates responses’.

One aspect of emotion definitions from emotion researchers that is largely absent from those of our respondents was the presence of a cognitive ‘appraisal’ component. This absence may be because, in the context of animal emotions, participants were hesitant to add such a conscious cognitive component (despite a large proportion of our participants attributing consciousness to at least some animal taxa). Interestingly, in Izard’s survey of emotion definitions [48], while appraisals and cognitive interpretations of a feeling were identified as ‘common aspects’ across the given definitions, they had the two lowest ranks of mean agreement rating of all the survey components included.

### 4.6. Limitations

Interpretation of our survey results is primarily constrained by two limitations. First, our sample size was somewhat small (*n* = 100 professional animal behaviour researchers) and was self-selected. This convenience sampling approach may have biased our sample towards individuals with relatively strong background knowledge or beliefs regarding animal emotions. It is possible that this bias could have resulted in individuals being more likely to participate if they believe that animals experience emotions, or strongly believe they do not, while others with less strong opinions may have chosen not to participate. While a limitation in our ability to survey the full spectrum of responses, we are confident that we have captured a large amount of variation in how scientist perceive animal emotions, similar to studies surveying public opinion. Importantly, all within-individual trends, which represent the bulk of our results, would be less affected by this potential selection bias.

Second, our survey asked respondents only about animal emotions generally: we did not instruct respondents to consider any specific emotions or categories of emotional states. A more detailed and specific assessment of particular emotional phenomena represents a rich opportunity for future research. Respondents’ assessment that ‘advanced cognitive abilities’ are necessary for the evolution of emotions suggests that they are not limiting their assessments only to fear responses or pain sensation, but this assumption is speculative on our part and requires more detailed future inquiry. We also cannot rule out that different respondents may have had different emotional states in mind when answering the questions.

### 4.7. Conclusions and optimism for future progress

Animal behaviour researchers who responded to our survey were optimistic regarding our ability to understand animal emotions and consciousness. Most respondents to our survey assessed understanding animal emotions as an important area of inquiry, with a large majority (71%) agreeing that animal behaviour, as a field, should do more to encourage investigation of ‘animal emotions, feelings, and consciousness’. Very large majorities asserted that animal emotions (90%) and consciousness (85%) can either be assessed using existing techniques or will be able to be assessed in the future given reasonable advances in technology.

The sum of our results indicates that respondents perceive (i) emotions to be taxonomically widespread and important in animals’ lives, (ii) measurable now or in the near future, and (iii) worthy of increased attention and research. This overall sentiment stands in stark contrast to the behaviourist sentiment of a century ago that emotions and consciousness represent either illusory or at best unmeasurable aspects of an animal’s life.

We share the optimism expressed by the respondents to our survey that progress regarding the distribution, functions, and limitations of animal emotions and consciousness will continue over the coming decades. We hope that this initial survey of sentiments and perceptions will serve as a baseline to which we or other researchers can compare in the future as new evidence emerges and our understanding regarding these questions becomes more precise.

## Data Availability

Data and code supporting analyses in this article are available from the Dryad Digital Repository [51]. Supplementary material is available online [52].
